# Mechanisms of ROS-induced mitochondria-dependent apoptosis underlying liquid storage of goat spermatozoa

**DOI:** 10.18632/aging.102295

**Published:** 2019-09-23

**Authors:** Tengfei Liu, Yawen Han, Ting Zhou, Ruihang Zhang, Hong Chen, Shulin Chen, Huiying Zhao

**Affiliations:** 1 College of Veterinary Medicine, Northwest A & F University, Yangling 712100, Shaanxi Province, China

**Keywords:** liquid storage, goat spermatozoa, mitochondria-dependent apoptosis, ROS, differentially expressed protein

## Abstract

Liquid storage of spermatozoa is important for artificial insemination and herd genetic breeding. However, the extended time of storage inducing the rapid decline in spermatozoa quality limits the development of this technology. The molecular mechanisms underlying liquid storage of spermatozoa remain largely unexplored. In this study, the effects of liquid storage on functional quality of spermatozoa were assessed in goat (*Capra hircus*). The time-dependent decline in spermatozoa motility showed a strong correlation with the significant increase in apoptosis. Moreover, apoptosis-related ultrastructural changes were observed, especially the defects in mitochondria. A significant decrease in mitochondrial membrane potential and changes in the expression of mitochondrial apoptosis-related proteins indicated mitochondrial dysfunction and mitochondrial apoptotic pathway activation. Notably, the abnormally high level of reactive oxygen species (ROS) caused by liquid storage resulted in oxidative damage to mitochondria and accelerated mitochondria-dependent apoptosis, as demonstrated by the addition of ROS scavenger N-acetylcysteine. Furthermore, critical differentially expressed proteins involved in mitochondria-dependent apoptosis and antioxidant defense were identified and profiled by quantitative proteomic analysis, facilitating the understanding of molecular regulation of ROS-induced mitochondria-dependent apoptosis. These outcomes provide insights into the mechanisms underlying liquid storage of goat spermatozoa and enhance the progress of semen storage technology.

## INTRODUCTION

Artificial insemination (AI) plays an essential role in goat breeding and is one of the most important means to improve the production traits and increase the rate of genetic progress [1–3]. Successful preservation of spermatozoa, as a vital technique of AI, would greatly increase the efficiency of AI [1]. Although cryopreservation can prolong the storage time of spermatozoa, the frozen-thawed spermatozoa possess the disadvantages of serious injuries in structure, poor fertilizing capacity and additional costs [4]. Liquid storage of spermatozoa at a reduced temperature is a practical alternative to cryopreservation when AI is performed within a relatively short time [3, 5]. Numerous studies are focusing on the preservation of spermatozoa utilizing liquid storage to improve the quality of spermatozoa in different species, such as the goat, boar and bull [2, 3, 6–8]. However, a rapid decline in the viability and fertilizing potential of spermatozoa during liquid storage usually occurs with the increase in storage time [3, 5], limiting the widespread use of this technology.

One of the major factors for the decline in viability and fertility is attributed to apoptosis in liquid-stored spermatozoa [5, 6]. Apoptosis is a physiologically programmed cell death mechanism that involves multiple cell death signaling and regulatory pathways. Earlier studies have considered that spermatozoa exhibit little transcriptional activity and, thus, do not undergo apoptosis [9–11]. Nevertheless, recent considerable evidences have strongly demonstrated the occurrence of apoptosis in spermatozoa, exhibiting signs of phosphatidylserine exteriorization, caspase activation, and DNA damage [11–14]. Clinical studies and laboratory findings have reported that apoptosis impacts the functional quality of spermatozoa [15, 16]. Spermatozoa in infertile men display apoptosis-like ultrastructural changes and the expression of typical markers of apoptosis [11, 16]. Furthermore, apoptosis seems to have a negative impact on the spermatozoa oocyte penetration potential, suggesting a negative association between apoptosis and spermatozoal fertility [17]. Remarkably, the activation of apoptosis is documented following the *in vitro* preservation of spermatozoa [7, 15, 18]. Studies in cryopreserved spermatozoa have observed the typical features of apoptosis, such as a decrease in the mitochondrial membrane potential and activation of caspases [18, 19]. Moreover, a time-dependent loss of viability and motility and alteration of membrane permeability in stored spermatozoa have been shown to exhibit a strong correlation with the induction of apoptosis [15, 20]. Additionally, the supplementation of specific inhibitors of apoptotic markers within the extender of preservation clearly improves the quality of spermatozoa [20, 21]. It is speculated that apoptosis may be a critical element to determine the storage of spermatozoa [7, 22, 23]. However, few studies have focused on the underlying mechanisms of apoptosis regulating the liquid storage of spermatozoa, especially in the goat.

Several extrinsic and intrinsic factors are responsible for apoptosis in spermatozoa. Reactive oxygen species (ROS), including superoxide anion (O_2_−∙), hydrogen peroxide (H_2_O_2_), and hydroxyl radical (∙OH), are considered a normal consequence of cellular metabolism in germ cells [24]. The physiological level of ROS exerts a vital function in the development and capacitation of normal germ cells [25, 26], whereas excessive production of ROS results in oxidative stress and apoptosis in spermatozoa [26, 27]. Liquid storage of spermatozoa was reported to disrupt the cellular oxidant-antioxidant balance, causing increased ROS production and directly leading to ROS-mediated damage to spermatozoa [6, 26]. Considerable studies have suggested that the over-production of ROS accelerates the process of apoptosis and induces the loss of spermatozoal functional competence [27–29], such as DNA damage, the loss of fertility, and change in the mitochondrial membrane architecture. Mitochondria, as the major site of intracellular ROS formation, are particularly susceptible to oxidative stress and mediate the intrinsic apoptotic pathway [30]. An abnormally high level of ROS can cause mitochondrial dysfunction, triggering the mitochondrial apoptotic pathway [24, 27, 29]. Moreover, spermatozoa mitochondria under preservation appear to be the most vulnerable cellular organelle to oxidative attack [31]. The defective function of preserved spermatozoa may be due to ROS-mediated mitochondrial damage and apoptosis [18, 19, 24]. However, the molecular roles of ROS-induced mitochondria-dependent apoptosis remain unclear in the liquid storage of goat spermatozoa.

The recent development of proteomics technologies has enabled the molecular studies of germ cells at the protein level and contributes to better understanding of the molecular mechanisms controlling spermatozoa quality and function [32, 33]. In this study, the occurrence of apoptosis was first assessed in liquid-stored spermatozoa of the goat (*Capra hircus*), and then the potential roles of ROS in inducing mitochondria-dependent apoptosis were explored. Furthermore, tandem mass tag (TMT)-based quantitative proteomic analysis was used to investigate the expression changes of critical proteins, facilitating uncovering the underlying molecular mechanisms of ROS-induced mitochondria-dependent apoptosis in the liquid storage of goat spermatozoa. The results of this study could provide rich insights into the improvement of semen storage technology and reproductive biotechnologies in the goat and other herd.

## RESULTS

### Assessment of goat spermatozoa motility and apoptosis during liquid storage

**Figure 1:**
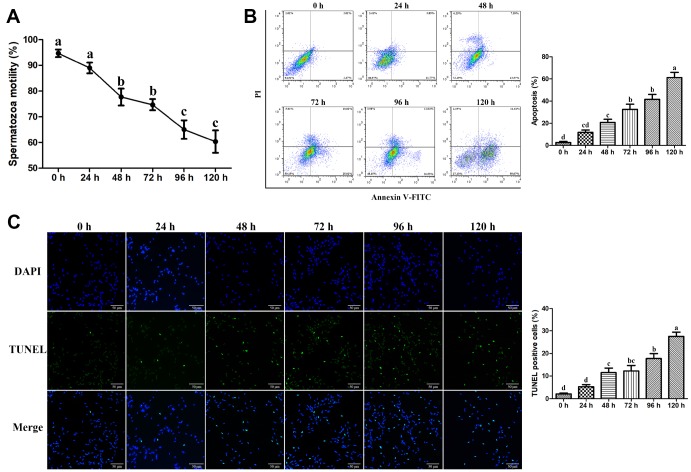
**Effects of liquid storage on the motility and apoptosis of goat spermatozoa.** (**A**) Spermatozoa motility was measured using Computer-Assisted Semen Analysis. The apoptosis rate was evaluated by the Annexin V-FITC/PI Apoptosis assay (**B**) and TUNEL staining (**C**) Scale bar = 50 μm. Values with different letters are significantly different from each other (*P* < 0.05).

The motility of goat spermatozoa declined gradually with the increase in the liquid storage time (Figure 1A). The initial motility of spermatozoa was more than 90% at 0 h of storage. A significant reduction (*P* < 0.05) of spermatozoa motility at 48 h and 72 h was detected compared with that at 0 h and 24 h under liquid storage. Similarly, the spermatozoa motility was significant decreased at 96 h and 120 h (*P* < 0.05). The obvious inflection points of spermatozoa motility alterations were observed at 48 h and 96 h, respectively. Additionally, when the storage duration was further prolonged (> 120 h), the spermatozoa motility was less than 60% (data not shown).

Flow cytometric analysis using Annexin V-FITC and PI double staining was performed to detect apoptosis in liquid-stored spermatozoa. The percentage of apoptotic spermatozoa was significantly higher at 48 h than that at 0 h and was gradually increased until 120 h (Figure 1B). Furthermore, TUNEL staining assays revealed that the number of TUNEL-positive spermatozoa was significantly increased until 120 h compared with that at 0 h with the increase in the liquid storage time (Figure 1C), suggesting the increase in apoptosis in liquid-stored spermatozoa. The time-dependent increase in apoptosis was closely related to the reduction of spermatozoa motility during liquid storage. Based on the results by assessing the spermatozoa motility and apoptosis, the stored spermatozoa at the three time points of 0 h, 48 h, and 96 h were selected and used for further studies.

### Ultrastructural changes in goat spermatozoa during liquid storage

**Figure 2:**
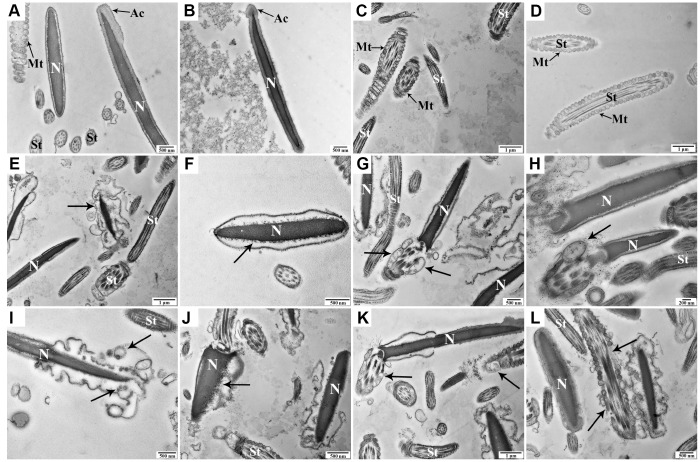
Transmission electron microscopy images of morphologic changes in goat spermatozoa at 0 h (**A**–**D**), 48 h (**E**–**H**), and 96 h (**I**-**L**) of liquid storage. (**A**, **B**) Spermatozoa exhibited the normal ultrastructure. (**C**, **D**) Morphologically intact mitochondria. (**E**) Membrane blebbing. (**F**) Nuclear envelope defects. (**G**, **H**) Mitochondrial swelling, vacuolation, and deformity. (**I**) Membrane blebbing and apoptotic body formation. (**J**) Nuclear fragmentation. (**K**, **L**) Mitochondrial swelling, vacuolation, deletion, and disordered arrangement. Nuclear (**N**), acrosome (Ac), spermatozoa tail (St), and mitochondria (Mt). Scale bar = 1 μm (**C**, **D**, **E**, **K**), 500 nm (**A**, **B**, **F**, **G**, **I**, **J**, **L**), and 200 nm (**H**).

The ultrastructural characteristics of spermatozoa were detected at 0 h, 48 h, and 96 h under liquid storage by transmission electron microscopy (TEM). Most spermatozoa exhibited a normal ultrastructure at 0 h, with an intact and smooth membrane that stayed tightly with the nuclear envelope (Figure 2A, 2B). Moreover, the complete nucleus was filled with evenly distributed chromatin, and the intact acrosome exhibited a smooth and complete acrosomal membrane and structure (Figure 2A, 2B). The mitochondria, which are tightly packed in the midpiece of spermatozoa, exhibited a clearly visible intact inner membrane, an outer membrane, and a well-defined intermembrane space (Figure 2C, 2D). With a prolonged storage time to 48 h and 96 h, some spermatozoa exhibited different types of apoptosis-related morphologic changes, such as plasma membrane blebbing (Figure 2E, 2I), apoptotic body formation (Figure 2I), defects in the nuclear envelope (Figure 2F), and nuclear fragmentation (Figure 2J). Notably, specific ultrastructural changes in the mitochondria, including swelling, vacuolation, deformity, and the partial absence or additional accumulations, were also observed (Figure 2G, 2H, 2K, 2L).

### Analysis of mitochondrial membrane potential (MMP) and expression changes in mitochondria-dependent apoptosis proteins

**Figure 3:**
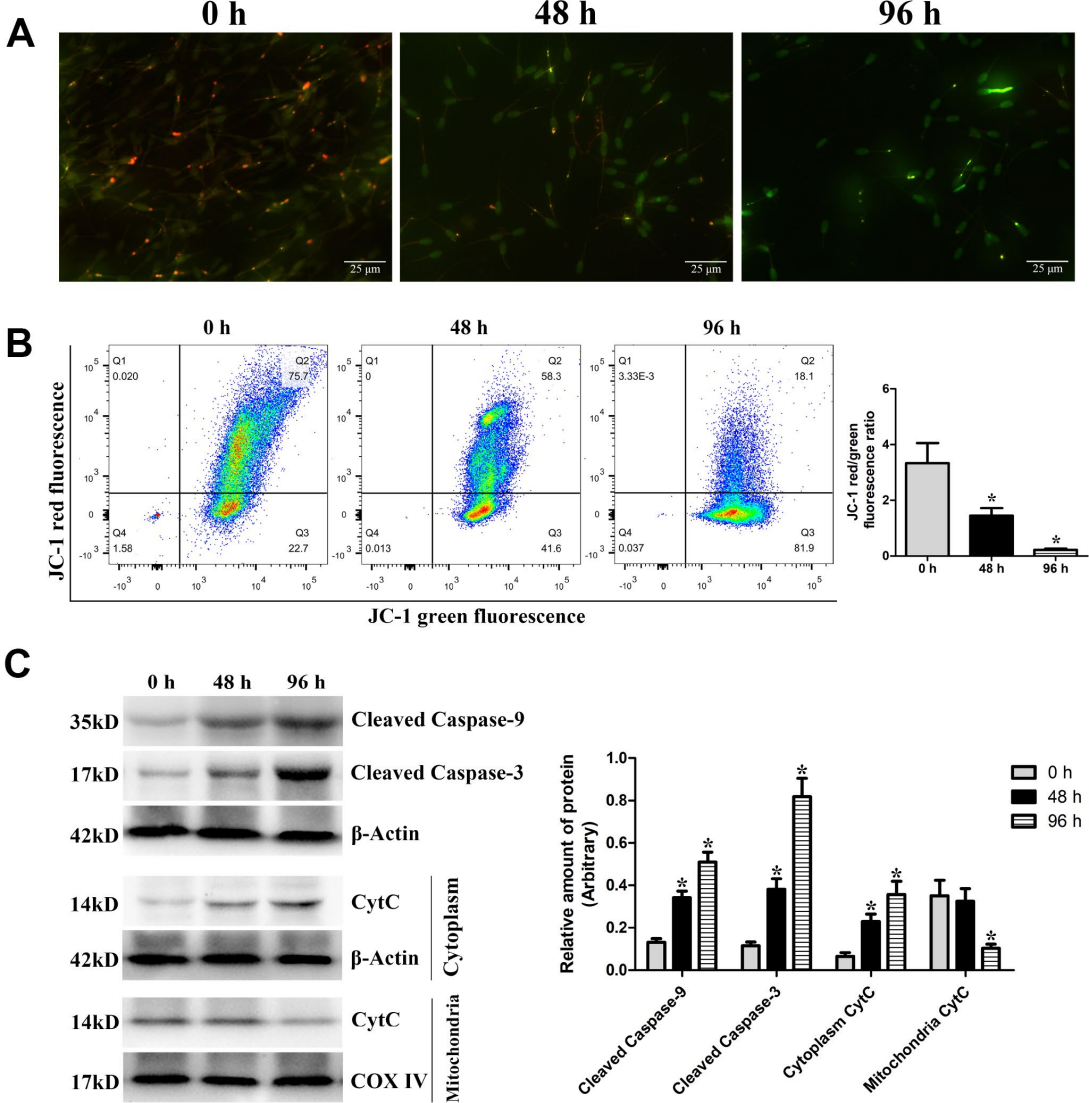
**Effects of liquid storage on the mitochondrial membrane potential (MMP) and expression changes in mitochondria-dependent apoptosis proteins in goat spermatozoa.** JC-1 staining with a fluorescence microscope (**A**) and flow cytometric analysis (**B**) showed change in the MMP. (**C**) Western blot analysis of Cleaved caspase-9, Cleaved caspase-3, and Cytochrome c (CytC) protein expressions in goat spermatozoa. *P < 0.05 compared to 0 h.

The effect of liquid storage on MMP in goat spermatozoa was measured using JC-1 staining. Compared with the control group (0 h), JC-1 aggregation (red) was decreased, and JC-1 monomer (green) was increased at 48 h and 96 h (Figure 3A), indicating the decline in MMP. Analysis by flow cytometry showed that the fluorescence ratio of red (JC-1 aggregates) to green (JC-1 monomers) was significantly reduced (*P* < 0.05) at 48 h and 96 h compared with that at 0 h (Figure 3B). Significant changes in MMP with prolonged storage time indicate that liquid storage causes the damage of mitochondria in spermatozoa. Furthermore, western blot analysis was performed to validate the relative levels of key proteins of the mitochondrial apoptotic pathway, such as Cleaved caspase-9, Cleaved caspase-3, and Cytochrome c (CytC). The protein levels of Cleaved caspase-9 and Cleaved caspase-3 in spermatozoa were significantly increased (*P* < 0.05) at 48 h and 96 h compared with those at 0 h (Figure 3C). The CytC level exhibited a significant increase (*P* < 0.05) in the cytoplasm at 48 h and 96 h compared with that at 0 h, as well as a decrease in the mitochondria. The results suggest that apoptosis in spermatozoa is upregulated with the increase in the liquid storage time and is involved in the mitochondrial apoptotic pathway.

### Analysis of the intracellular ROS levels and mitochondrial antioxidant enzyme activities

**Figure 4:**
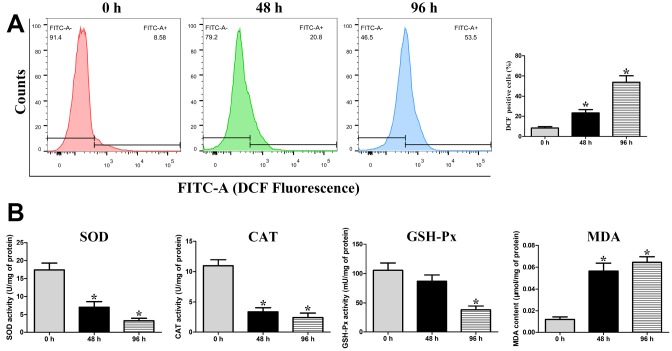
Assessment of intracellular ROS levels (**A**), and the activities of mitochondrial SOD, CAT, and GSH-Px enzymes and the concentration of MDA in the mitochondria of liquid-stored goat spermatozoa (**B**). *P < 0.05 compared to 0 h.

The intracellular ROS was detected using the fluorescent probe DCFH-DA. The percentage of DCF-positive cells (increase of fluorescence) was significantly increased (*P* < 0.05) at 48 h (23.27%) and 96 h (53.83%) compared with that at 0 h (8.36%), suggesting that liquid storage enhances ROS generation in spermatozoa (Figure 4A). Superoxide dismutase (SOD), catalase (CAT), and glutathione peroxidase (GSH-Px) are three important enzymes in the antioxidant defense system, while malondialdehyde (MDA) is regarded as a major marker of lipid peroxidation in spermatozoa. The activities of SOD, CAT, and GSH-Px, as well as the content of MDA, were evaluated in the mitochondria of liquid-stored spermatozoa (Figure 4B). A similar variation trend among the SOD, CAT, and GSH-Px activities was observed at different time points of liquid storage. The levels of SOD and CAT were significantly decreased (*P* < 0.05) at both 48 h and 96 h compared with those at 0 h, and the level of GSH-Px was significantly decreased (*P* < 0.05) at 96 h. A significant increase (*P* < 0.05) in the MDA level was detected both at 48 h and 96 h compared with that at 0 h. These results suggest that the increase in ROS induced by liquid storage results in the damage to mitochondria in goat spermatozoa.

### Validation of ROS-induced mitochondria-dependent apoptosis during the liquid storage of goat spermatozoa

**Figure 5:**
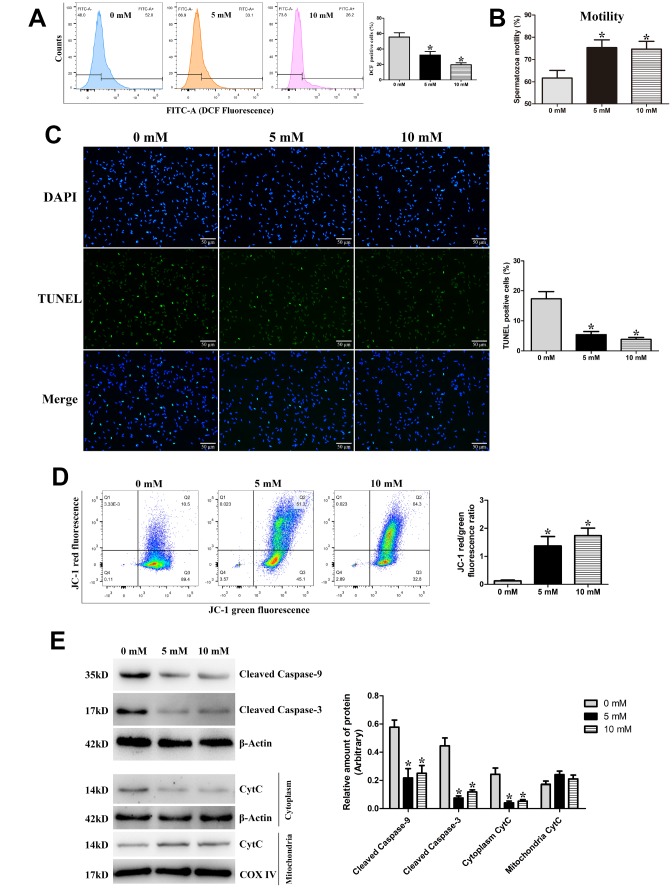
**Validation of ROS-induced mitochondria-dependent apoptosis during the liquid storage of goat spermatozoa by the addition of the ROS scavenger NAC (0 mM, 5 mM, and 10 mM) at 96 h of storage.** (**A**) Change in the intracellular ROS levels. (**B**) Assessment of spermatozoa motility. (**C**) Evaluation of the apoptosis rate by TUNEL staining. (**D**) Change in the MMP by JC-1 staining. (**E**) Protein expression of Cleaved caspase-9, Cleaved caspase-3, and Cytochrome c (CytC) by western blot analysis. *P < 0.05 compared to 0 mM.

To further validate the relationship between excess ROS production and mitochondria-dependent apoptosis during the liquid storage of goat spermatozoa, the ROS scavenger N-acetylcysteine (NAC) with different concentrations (0, 5, and 10 mM) was added to the semen extenders and the ROS level was detected at 96 h of storage. Compared with the control group (0 mM NAC), the percentage of DCF-positive cells (intracellular ROS level) was significantly decreased (*P* < 0.05) in the 5 mM and 10 mM NAC groups in a dose-dependent manner (Figure 5A). Meanwhile, the motility of goat spermatozoa was significantly increased (*P* < 0.05) in the 5 mM and 10 mM NAC groups (Figure 5B). Moreover, TUNEL staining analysis showed that the rate of apoptosis in stored spermatozoa was significantly decreased (*P* < 0.05) in the 5 mM and 10 mM NAC groups (Figure 5C). JC-1 staining analysis showed that MMP was significantly increased (*P* < 0.05) in the 5 mM and 10 mM NAC groups (Figure 5D). Additionally, protein expression by western blot analysis showed that the levels of Cleaved caspase-9 and Cleaved caspase-3 proteins were significantly decreased (*P* < 0.05) in the 5 mM and 10 mM NAC groups (Figure 5E). Meanwhile, the CytC protein level exhibited a significant decrease in the cytoplasm (Figure 5E). The decrease in apoptosis, increase in MMP, and expression changes in mitochondrial apoptosis-related proteins were coincident with the inhibition of intracellular ROS in the NAC treatment groups, indicating that the excess generation of ROS stimulates the mitochondrial apoptotic pathway in liquid-stored goat spermatozoa.

### Identification of differentially expressed proteins (DEPs) by proteomic analysis

**Figure 6:**
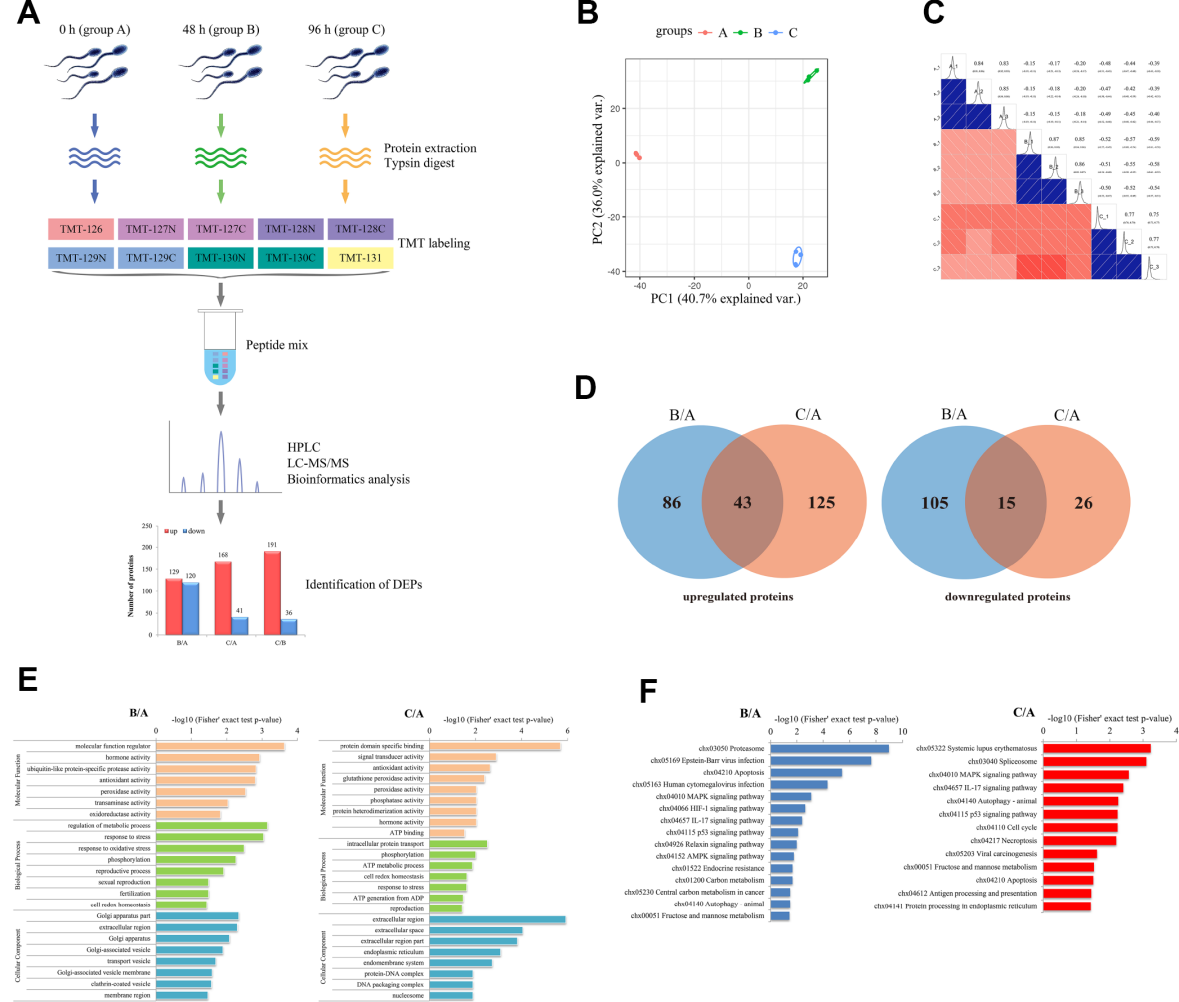
**Characterization of differentially expressed proteins (DEPs) involved in the liquid storage of goat spermatozoa by TMT-based quantitative proteomic analysis.** (**A**) The workflow of proteomic analysis and identification of DEPs. B/A, 48h/0h; C/A, 96h/0h; C/B, 96h/48h. (**B**) Analyses of principal component analysis (PCA) in different replicated protein samples. (**C**) Analyses of Pearson correlation coefficients (PCC) in different replicated protein samples. (**D**) Venn diagrams of DEPs in the B/A and C/A comparisons. (**E**) GO enrichment analysis of DEPs in the B/A and C/A comparisons. (**F**) KEGG enrichment analysis of DEPs in the B/A and C/A comparisons.

In this study, TMT-based quantitative proteomic analysis was performed to identify differentially expressed proteins (DEPs) that regulate the liquid storage of goat spermatozoa. Samples at three different storage times, including 0 h (group A), 48 h (group B), and 96 h (group C), were separately used for analyses (Figure 6A). In total, 2,779 proteins with 2,336 quantified proteins were identified in the three sample groups of spermatozoa. The reliability of data from the replicated samples was validated through principal component analysis (PCA) and Pearson correlation coefficients (PCC) analyses (Figure 6B, 6C). After comparing the protein expression levels and filtering by a threshold value of a fold change > 1.5 or < 0.67 and a *p*-value < 0.05, 129, 168, and 191 upregulated proteins and 120, 41, and 36 downregulated proteins were identified in B/A, C/A, and C/B comparisons (Figure 6A), respectively. The number of upregulated proteins was obviously more than that of downregulated proteins in the three comparisons. The Venn diagrams of the identified DEPs showed that 43 upregulated and 15 downregulated proteins were commonly identified in both B/A and C/A comparisons (Figure 6D).

To further explore the biological functions of the identified DEPs, functional enrichment analysis was performed based on Gene Ontology (GO) categories and Kyoto Encyclopedia of Genes and Genomes (KEGG) pathways. The results showed that these identified DEPs were enriched in 23 (B/A) and 24 (C/A) GO categories (Figure 6E). Several GO terms were related to the process of reproductive development, such as ‘reproductive process’ (GO: 0022414), ‘sexual reproduction’ (GO: 0019953), ‘fertilization’ (GO: 0009566), and ‘reproduction’ (GO: 0000003). Moreover, specific GO terms, such as ‘antioxidant activity’ (GO: 0016209), ‘cell redox homeostasis’ (GO: 0045454), ‘oxidoreductase activity’ (GO: 0016684), and ‘response to stress’ (GO: 0006950), were involved in the stress response and antioxidant system (Figure 6E). KEGG pathway analysis showed that these identified DEPs were assigned to 15 (B/A) and 13 (C/A) pathways (Figure 6F). Several enriched KEGG pathways were involved in the process of cell death, such as ‘MAPK signaling pathway’ (chx04010), ‘Necroptosis’ (chx04217), ‘p53 signaling pathway’ (chx04115), and ‘Apoptosis’ (chx04210), which may participate in the control of spermatozoa survival and quality (Figure 6F).

### Analysis of critical DEPs regulating the liquid storage of spermatozoa

**Figure 7:**
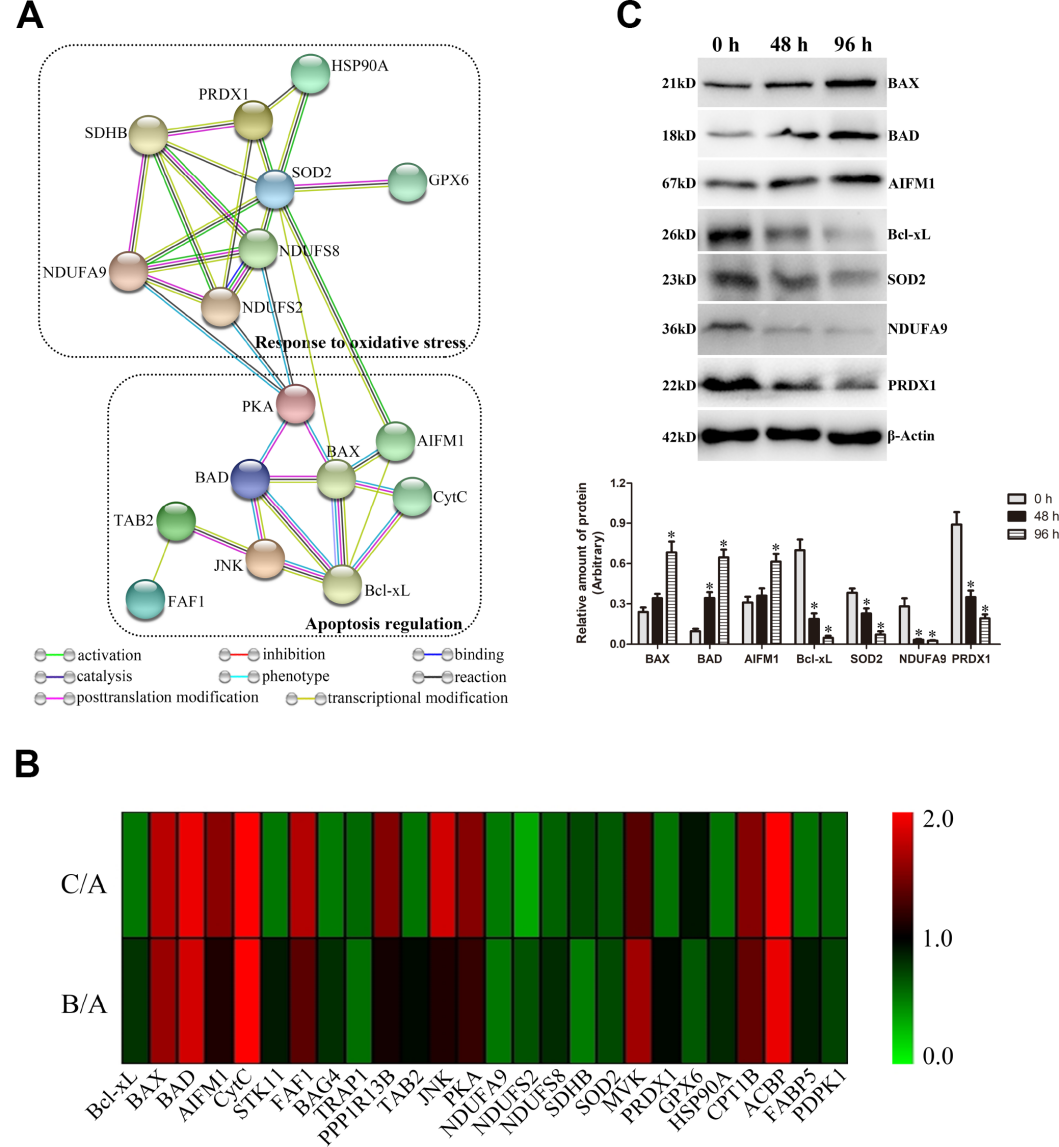
**Validation of critical differentially expressed proteins (DEPs) involved in apoptosis regulation and oxidative stress in goat spermatozoa.** (**A**) Protein-protein interaction analysis of critical DEPs. (**B**) Heat map of the protein expression of critical DEPs. (**C**) Western blot analysis of critical DEP expressions. *P < 0.05 compared to 0 h.

To investigate the critical DEPs regulating the liquid storage of goat spermatozoa, the putative functions of DEPs were analyzed based on the results of annotation and functional classification. In total, 26 critical DEPs were identified and mainly involved in vital biological processes, such as the apoptosis regulation and oxidative phosphorylation (Table 1). The protein-protein interaction analysis of critical DEPs revealed that eight interacted proteins were involved in the response to oxidative stress, and nine interacted proteins were involved in the processes of apoptosis regulation (Figure 7A). These critical proteins may play important roles in the molecular regulation of liquid storage in goat spermatozoa.

**Table 1:** Critical DEPs involved in the liquid storage of goat spermatozoa.

**Protein ID**	**Name**	**Description**	**B/A, type**	**C/A, type**
apoptosis-related protein				
XP_005686508.1	Bcl-xL	bcl-2-like protein 1	0.799, no	0.529, down
XP_017918239.1	BAX	BCL2 associated X, apoptosis regulator	1.602, up	1.728, up
XP_017898279.1	BAD	BCL2 associated agonist of cell death	1.832, up	1.934, up
XP_005700436.1	AIFM1, PDCD8	apoptosis-inducing factor 1, mitochondrial isoform X2	1.127, no	1.546, up
XP_005675975.1	CytC	cytochrome c 2	2.259, up	2.427, up
XP_017905952.1	STK11, LKB1	serine/threonine-protein kinase STK11 isoform X3	0.901, no	0.531, down
XP_005678500.1	FAF1	FAS-associated factor 1	1.358, no	1.707, up
XP_017897284.1	BAG4	BAG family molecular chaperone regulator 4	0.818, no	0.519, down
XP_017895731.1	TRAP1, HSP75	heat shock protein 75 kDa, mitochondrial isoform X2	0.563, down	0.592, down
XP_017921573.1	PPP1R13B, ASPP1	apoptosis-stimulating of p53 protein 1 isoform X2	1.081, no	1.517, up
MAPK signaling pathway				
XP_017908818.1	TAB2, MAP3K7IP2	TGF-beta-activated kinase 1 and MAP3K7-binding protein 2	0.961, no	0.537, down
XP_017907112.1	JNK	mitogen-activated protein kinase 9 isoform X6	1.129, no	1.825, up
XP_017906682.1	PKA	cAMP-dependent protein kinase catalytic subunit alpha	1.214, no	1.526, up
Oxidative phosphorylation				
XP_005681037.1	NDUFA9	NADH dehydrogenase [ubiquinone] 1 alpha subcomplex subunit 9	0.529, down	0.519, down
XP_013831079.1	NDUFS2	NADH dehydrogenase [ubiquinone] iron-sulfur protein 2	0.678, no	0.341, down
XP_017898410.1	NDUFS8	NADH dehydrogenase [ubiquinone] iron-sulfur protein 8	0.829, no	0.619, down
XP_017910172.1	SDHB	succinate dehydrogenase [ubiquinone] iron-sulfur subunit	0.508, down	0.713, no
Peroxisome				
XP_017908917.1	SOD2	superoxide dismutase	0.712, no	0.652, down
XP_017916592.1	MVK	mevalonate kinase	1.635, up	1.347, no
XP_005678591.1	PRDX1	peroxiredoxin-1	0.973, no	0.549, down
XP_005696754.2	GPX6	glutathione peroxidase 6	0.651, down	0.923, no
XP_017921728.1	HSP90A	heat shock protein HSP 90-alpha	0.845, no	0.534, down
PPAR signaling pathway				
XP_017904483.1	CPT1B	carnitine O-palmitoyltransferase 1	1.396, no	1.528, up
XP_005676309.1	ACBP	acyl-CoA-binding protein	1.927, up	2.305, up
XP_017913867.1	FABP5	fatty acid-binding protein	0.887, no	0.551, down
XP_017895849.1	PDPK1	3-phosphoinositide-dependent protein kinase 1	0.741, no	0.601, down

Most DEPs were significantly differentially expressed in the comparisons of B/A and C/A, exhibiting a time-dependent pattern (Table 1, Figure 7B). Notably, several DEPs belonging to Bcl-2 family proteins were identified in this study: Bcl-xL was downregulated, and BAX and BAD were upregulated. Moreover, critical proteins (such as CytC and AIFM1), as the known promoter of apoptosis, were upregulated in the comparisons of B/A and C/A. Additionally, significantly downregulated levels of NDUFA9, NDUFS2, and NDUFS8 proteins, belonging to the subunits of Complex I in mitochondrial electron transport chain (ETC), as well as those of a major Complex II subunit SDHB, were detected. The protein expression levels of these critical DEPs were further validated by western blot analysis (Figure 7C). Most selected proteins exhibited similar expression profiles to that by TMT analysis, suggesting the dependability of results by proteomic analysis.

## DISCUSSION

### The ROS-induced mitochondrial apoptotic pathway is implicated in the liquid storage of goat spermatozoa

The development of semen storage technology offers more opportunities for the successful application of AI in herd breeding [1, 2]. Liquid storage of spermatozoa is a practical and effective method to minimize the spermatozoa injuries that is generated seriously by the freezing and thawing method [1]. In recent decades, many studies have tried to prolong the time of liquid storage through optimizing the environmental conditions, greatly improving the *in vitro* viability and fertilizing potential of spermatozoa [2, 3, 5, 8]. In fact, the results of liquid storage remain unsatisfactory in many species, especially in the goat. An inevitable consequence of liquid storage is the rapid deterioration of spermatozoa quality, when the period of storage is extended [3, 5]. Previous studies reported that long term liquid storage of ram semen caused significant decrease of spermatozoa motility [2, 3, 5, 34]. Similarly, in this study, the motility of liquid-stored goat spermatozoa was assessed and showed a significant decrease with the increase in storage time. Various extrinsic and intrinsic factors influence the storage of spermatozoa. Nevertheless, the underlying mechanisms involved in the liquid storage of spermatozoa remain largely unexplored.

Increasing numbers of studies have demonstrated that apoptosis, as an ongoing physiological phenomenon, seems to exert a strong influence on the decrease in spermatozoa quality [5, 6]. In stored spermatozoa, apoptosis may directly affect the changes in typical spermatozoa quality parameters, including motility, acrosome status, membrane integrity, and phosphatidylserine distribution [14–16]. Previous studies revealed that freezing and thawing-induced apoptosis is inversely correlated with spermatozoa motility and plasma membrane integrity during cryopreservation [15, 19, 22]. Moreover, for liquid storage, the increase in apoptosis exhibiting a significant relationship with the decrease in spermatozoa quality was reported in many species, such as the boar, bull, and stallion [6–8, 35]. In the current study, the occurrence of apoptosis in liquid-stored goat spermatozoa was detected both by TUNEL staining and Annexin-V-FITC/PI staining, which are considered as routine and effective methods to validate spermatozoa undergoing apoptosis and have been utilized in many similar studies [36, 37]. Similar to previous studies [3, 7], the rate of apoptosis exhibited a significant time-dependent increase and was significantly related to the reduction of spermatozoa motility, indicating that apoptosis negatively affects the liquid storage of goat spermatozoa. Additionally, similar results suggested that the reduction of apoptosis can cause significant enhancement of spermatozoa motility and viability [20, 21]. Hence, apoptosis may be a critical mechanism involved in the liquid storage of spermatozoa and can be considered a useful index to assess the spermatozoa quality in the goat.

Notably, in this study, the occurrence of apoptosis was accompanied by ultrastructural changes in spermatozoa, including plasma membrane blebbing, apoptotic body formation, defects in the nuclear envelope, and nuclear fragmentation. The defined signs of apoptosis were similar to the previous observations in human spermatozoa [11], further providing evidence for liquid-stored spermatozoa undergoing apoptosis. Particularly, the distinct alteration of the mitochondrial ultrastructure was observed in the current study. It was reported that structural defects in mitochondria represent a main feature of mitochondrial dysfunction and may be strongly associated with mitochondria-dependent apoptosis [38, 39]. Previous studies demonstrated that changes in the mitochondrial structure can be used as early apoptotic markers in human spermatozoa [11, 40]. Our morphological observations implied that the mitochondrial apoptotic pathway may be implicated in the liquid storage of goat spermatozoa. Moreover, further assessments of the MMP and expression levels in mitochondrial apoptosis-related proteins were employed to confirm the speculation. As expected, analysis by JC-1 staining showed a significant decline in MMP, which exhibits an early sign of the intrinsic mitochondrial pathway leading to apoptosis [41]. Western blot analysis also showed changes in the levels of critical proteins involved in the mitochondrial apoptotic pathway, including significant increases in Cleaved caspase-9 and Cleaved caspase-3 and the large release of CytC from the mitochondria to the cytoplasm. These molecular evidences clearly indicate that liquid storage results in activation of the intrinsic mitochondrial apoptotic pathway in goat spermatozoa.

Numerous reports have suggested that an imbalance of antioxidant defenses usually follows the impairment of mitochondrial function, which is responsible for mitochondria-dependent apoptosis [27, 42]. Oxidative stress by ROS seems to significantly contribute to the damage to mitochondria and apoptosis of spermatozoa [3, 26, 42]. Studies in semen disorders have demonstrated the putative vital roles of ROS in inducing mitochondrial apoptotic signaling [43]. He et al. [44] reported that alleviating ROS-driven mitochondrial dysfunction can inhibit the apoptosis of germ cells. Falch et al. [34] found a gradual increase of ROS production throughout the 96 h of liquid storage of ram semen. In this study, the endogenous ROS level in goat spermatozoa was significantly increased with the extension of storage time, which is similar to the previous result [34], and the decrease in the mitochondrial SOD, CAT, and GSH-Px levels was also detected, indicating that liquid storage induced the excessive generation of ROS and decrease in antioxidant properties. It is well known that high levels of ROS cause lipid peroxidation of plasma membranes, resulting in alteration of spermatozoa functional quality [39, 45]. Previous study in boar spermatozoa revealed that an increase in lipid peroxidation was associated with decrease in motility and viability [6]. The occurrence of lipid peroxidation in mitochondria adversely affects mitochondrial integrity and function, which is one of major factors to explain the reduction of spermatozoa motility [6, 46]. Importantly, lipid peroxidation causes the loss of MMP, directly inducing the release of apoptotic factor CytC protein and then activating apoptosis in spermatozoa [39, 46]. In the present study, the concentration of mitochondrial MDA was significantly increased, suggesting that lipid peroxidation was significantly accelerated by liquid storage and resulted in the damage of mitochondria. Moreover, our observations of alteration of MDA were closely associated with the above results on mitochondrial structural alterations and expression changes of mitochondrial apoptotic proteins, implying that the long period of liquid storage causes ROS-mediated oxidative stress that is associated with mitochondria-dependent apoptosis. More importantly, our further analysis by inhibition of ROS validates the axis of the regulatory process. In the current study, the supplementation of the ROS scavenger NAC obviously suppressed the generation of ROS in a dose-dependent manner and enhanced the spermatozoa motility, followed by apoptosis inhibition, increase in MMP, and decrease in Cleaved caspase-9, Cleaved caspase-3, and CytC protein levels. Taken together, these findings support the conclusion that liquid storage causes an abnormal high level of ROS, resulting in mitochondrial damage, which then stimulates mitochondria-dependent apoptosis in goat spermatozoa [27, 43].

### Putative molecular mechanism of ROS-induced mitochondria-dependent apoptosis in goat spermatozoa during liquid storage

Considerable evidences have demonstrated the prominent role of mitochondria-dependent apoptosis in germ cells [39, 47, 48]; however, the molecular basis and underlying mechanism of ROS-induced mitochondria-dependent apoptosis has not been clearly explored in spermatozoa during liquid storage. In this study, TMT-based quantitative proteomic analysis was used to profile the critical proteins involved in the mitochondrial apoptotic pathway during the liquid storage of goat spermatozoa. It is well known that Bcl-2 family proteins are the essential sentinels of the mitochondrial apoptotic pathway to control the first regulatory step of mitochondria-dependent apoptosis [49]. Studies have revealed that the coordinated expression levels of pro- and anti-apoptotic Bcl-2 family proteins ultimately decide germ cell apoptosis [48–50]. Our studies by proteomic analysis identified several DEPs belonging to the Bcl-2 family, such as the downregulated Bcl-xL (anti-apoptotic protein) and upregulated BAX and BAD (pro-apoptotic proteins), suggesting that these Bcl-2 family proteins may play pivotal roles in the mitochondrial pathway of apoptosis. Upon apoptotic stress, the interplay of pro- and anti-apoptotic members of Bcl-2 family regulates the mitochondrial apoptotic pathway through controlling the permeabilization of the outer mitochondrial membrane (OMM) and subsequent release of CytC into the cytoplasm to activate the caspase cascade [39, 50, 51]. Importantly, in the current study, both proteomic analysis and western blot analysis showed the upregulation of CytC protein, which co-occurred with the induction of the mitochondrial permeability transition, strongly indicating the activation of mitochondrial apoptotic pathway [41]. Additionally, AIFM1, a downstream molecule of the mitochondrial apoptotic pathway, also exhibited significant upregulation. Previous studies have shown that AIFM1 can induce the release of the mitochondrial protein CytC, activate caspase proteins and then induce apoptosis [52]. Moreover, the increase in cytoplasmic AIFM1 promotes the release of more AIFM1 from the mitochondria, further accelerating apoptosis [53]. The expression alterations of these apoptosis-related proteins may initiate mitochondria-dependent apoptosis during the liquid storage of goat spermatozoa.

Oxidative stress induced by excessive ROS generation is indispensable for mitochondria-dependent apoptosis [3, 26, 42]. The inner mitochondrial membrane (IMM) includes multiple complexes that make up mitochondrial ETC, which promotes ROS production [39]. Previous comparative proteomic analysis has suggested that the expression of subunits in ETC complexes was altered in spermatozoa exposed to excessive oxidative stress [54, 55]. Inhibition of mitochondrial complex I can induce a further increase in ROS production, eventually leading to apoptosis [56]. In the present study, several DEPs, including NDUFA9, NDUFS2, and NDUFS8, were involved in ETC complex I and exhibited significant downregulation during liquid storage, indicating the defects in mitochondrial complex I. Additionally, our study identified a downregulated SDHB protein, which is a key subunit of mitochondrial complex II and has evolved a role in apoptosis induction [57]. These subunits with decreased expression may be attributed to excess ROS generation and are potential contributors to mitochondria-dependent apoptosis in liquid-stored goat spermatozoa. Moreover, the effective scavenging of ROS is an essential process to protect germ cells from oxidative stress [39, 58]. In this study, several proteins related to ROS scavenging, such as SOD2 and PRDX1, were markedly repressed, and may contribute to antioxidant system disorders. The low expression of SOD2 protein agrees with our above results on the decreased enzyme activity of SOD. Additionally, PRDX1 is regarded as an antioxidant and functions in reducing ROS and inhibiting cell apoptosis [59]. In general, these differentially regulated proteins involved in the antioxidant defense system disturb the balance between ROS generation and scavenging, directly triggering the mitochondrial apoptotic pathway in goat spermatozoa.

Furthermore, ROS-activated MAPK signaling regulating the progression of apoptosis complicates the mechanisms of ROS-induced apoptosis [58, 60, 61]. Previous studies have shown that increased ROS, a vital second messenger, could activate MAPK signaling, which mediated the regulation of many biological processes in spermatozoa [60–62]. Moreover, the ROS-activated MAPK signaling pathway contributes to expression changes in Bcl-2 family proteins, leading to activation of the mitochondrial apoptotic pathway [63, 64]. As expected, in the present study, functional enrichment analysis showed that the ‘MAPK signaling pathway’ (chx04010) was significantly enriched. Being similar to the differential expression of critical Bcl-2 family proteins, several DEPs, including TAB2, JNK, and PKA, were identified and involved in the MAPK signaling pathway. These results implied another critical mechanism that ROS, as a second messenger, may regulate mitochondria-dependent apoptosis by modulating the MAPK signaling cascade during the liquid storage of goat spermatozoa, which clearly require further researches.

## CONCLUSIONS

Liquid storage causes the decrease in the functional quality of spermatozoa in the goat. Notably, a significant increase in apoptosis strongly affected the storage of spermatozoa, which was implicated in the regulation of the intrinsic mitochondrial apoptotic pathway. Moreover, excessive generation of intracellular ROS led to oxidative damage to mitochondria and accelerated mitochondria-dependent apoptosis. These findings indicated that ROS-induced mitochondria-dependent apoptosis is of great importance. Furthermore, TMT-based quantitative proteomic analysis identified critical DEPs involved in mitochondria-dependent apoptosis and antioxidant defense, which will contribute to uncover the molecular regulatory mechanisms underlying the liquid storage of goat spermatozoa.

## MATERIALS AND METHODS

### Ethics statement

In this study, all experimental procedures and animal care were conducted according to the guidelines of the Animal Research Institute Committee (Northwest A & F University, Shaanxi, China). The protocol was approved by the Science and Technology Agency of Shaanxi Province under permit NO. SYXK (SN) 2016-004, and all efforts were made to minimize animal suffering.

### Semen collection and liquid storage

Semen samples were collected from six mature Guanzhong dairy goats (2-3 years of age) using an artificial vagina. The initial semen quality was assessed for each ejaculate: only semen with a volume > 0.5 mL, spermatozoa concentration > 3 × 10^9^/mL, and normal motility > 90% was accepted. All acceptable semen was diluted to a final spermatozoa concentration of 3 × 10^8^/mL with a pre-warmed Tris-based extender (Tris: 3.63 g/100 mL; fructose: 0.50 g/100 mL; citric acid: 1.99 g/100 mL; egg yolk: 10 mL/100 mL; penicillin: 5000 IU/100 mL; streptomycin: 0.1 g/100 mL; pH 6.8) as previously described [2]. For the experiments of ROS inhibition, NAC (ROS scavenger) was added to the semen extender at the concentrations of 0 (control), 5, and 10 mM, respectively. The diluted semen was allowed to adapt to room temperature for 30 min and was maintained in the refrigerator at 4 °C.

### Evaluation of spermatozoa motility

A computer-assisted semen analysis (CASA) system (HTM-IVOS 12, Hamilton Thorne, Beverly, USA) was used to assess spermatozoa motility with default parameters as previously described [65]. Briefly, a pre-warmed chamber slide was loaded with 3 μL of diluted sample (concentration of 3 × 10^6^/mL) at 37 °C and was observed using a Nikon phase contrast microscope (TE-2000U, Nikon Co., Tokyo, Japan) at 200× magnification. Five fields of each group of samples were captured, and at least 1,000 spermatozoa were counted. The motility of spermatozoa that were stored at 4 °C was examined at 0 h and every 24 h of storage thereafter. The preservation time with spermatozoa motility above 60% was recorded as the effective survival period.

### Evaluation of apoptosis

An Annexin V-FITC apoptosis detection kit (ab14085, Abcam Inc., Cambridge, USA) was employed to quantify the changes of apoptosis in spermatozoa according to the manufacturer’s instructions. Briefly, 1 × 10^6^ spermatozoa were washed in PBS, resuspended in 500 μL of binding buffer, and stained with 5 μL of Annexin V-FITC and 5 μL of PI at room temperature for 5 min in the dark. After incubation, the samples were centrifuged (800 × *g*, 10 min, 4 °C) and the pellet was resuspended with binding buffer. The apoptosis of spermatozoa was analyzed by flow cytometry (FACS Calibur, BD Biosciences, San Jose, USA). The amount of DNA fragmentation was determined by a terminal deoxynucleotidyl transferase-mediated nick end labeling (TUNEL) assays kit (S7111, Millipore, Billerica, USA) according to the manufacturer’s instructions. Briefly, the fixed spermatozoa on the slides were permeabilized with 0.1% (v/v) Triton X-100 for 2 min on ice and then were incubated in 50 μL of the TUNEL reaction mixture for 1 h at 37 °C in the dark. For the negative control, slides were incubated with the TUNEL reagent in the absence of TdT enzyme. The nuclei were counterstained using 4’-6-diamidino-2-phenylindole (DAPI; Roche Diagnostics, Basel, Switzerland). The percentage of apoptotic cells was calculated as a ratio of the number of TUNEL-positive cells to the total number of DAPI-positive cells. Five fields per section were randomly examined at 400× magnification under a fluorescence microscope (BX51; Olympus, Tokyo, Japan).

### Transmission electron microscopy (TEM)

Spermatozoa were fixed with 2% glutaraldehyde in 0.2 M PBS (pH 7.4) at 4 °C for 48 h and were centrifuged for 10 min at 800 × *g*. The pellet was then post-fixed in ice-cold 1% OsO_4_ for 2 h. The samples were dehydrated in increasing ethanol concentrations and were embedded in epoxy resin. Ultrathin sections (50 nm thick) were mounted on copper grids, counterstained with Reynolds lead citrate and examined with a Tecnai G2 Spirit TEM (FEI, Czech Re-public) at 80 kV.

### Measurement of MMP

The spermatozoa MMP was determined using the dye 5,5′,6,6′-tetrachloro-1,1′,3,3′-tetraethylbenzimidazol carbocyanine iodide (JC-1; ab113850, Abcam Inc., Cambridge, USA). Briefly, 1 × 10^6^ spermatozoa were washed in dilution buffer and were stained with 10 μM JC-1 for 30 min at 37 °C. The spermatozoa were washed again with dilution buffer, analyzed by flow cytometry (FACS Calibur, BD Biosciences, San Jose, USA) and imaged by fluorescence microscopy (BX51; Olympus, Tokyo, Japan). Spermatozoa emitting green fluorescence (JC-1 monomer) indicated low MMP, and spermatozoa emitting red fluorescence (JC-1 aggregation) indicated high MMP.

### Western blot analysis

Western blotting was performed using equal amounts of protein (50 μg/lane) from the stored spermatozoa. The total proteins of spermatozoa were homogenized in ice-cold RIPA buffer. The cytosolic and mitochondrial proteins were isolated from spermatozoa according to the protocols of a commercial Mitochondria Isolation Kit (89801, Thermo Scientific, Rockford, USA). Specific primary antibodies to Cleaved caspase-9 (ab2324), Cleaved caspase-3 (ab49822), CytC (ab90529), *β*-Actin (ab8227), COX IV (ab16056), Bax (ab53154), Bad (ab32445), AIFM1 (ab99437), Bcl-xL (ab32370), SOD2 (ab13533), NDUFA9 (ab128744), and PRDX1 (ab41906) were obtained from Abcam, Inc. (Cambridge, MA, USA). The samples were incubated at 4 °C overnight with the corresponding primary antibodies and subsequently with peroxidase-linked donkey anti-rabbit IgG (ab6802; Abcam Inc., Cambridge, MA, USA) for 2 h. An enhanced chemiluminescence (ECL) solution was used to visualize the target bands, and Quantity One software (Bio-Rad Laboratories) was employed to measure the relative band intensities.

### Detection of the intracellular ROS levels and activities of mitochondrial antioxidant enzymes

The level of intracellular ROS in spermatozoa was measured using the fluorescent probe redox-sensitive-fluoroprobe-2′,7′-dichlorofluorescein-diacetate (DCFDA; D6883, Sigma-Aldrich, St. Louis, USA). Briefly, 1 × 10^6^ spermatozoa were washed in PBS and were incubated with 100 mM DCFDA at 37 °C for 30 min. The fluorescence of DCF was measured on a flow cytometer at a wavelength of 485/535 nm.

Mitochondrial protein was isolated from liquid-stored spermatozoa using a Mitochondria Isolation Kit (89801, Thermo Scientific, Rockford, USA) according to the manufacturer’s recommendations. The concentration of mitochondrial protein was determined using a BCA protein assay kits (23225, Thermo Fisher Scientific, Rockford, USA). The activities of mitochondrial SOD, CAT, and GSH-Px were measured using commercial assay kits (A001-3, A007-1, A005; Nanjing Jiancheng Bioengineering Institute, China) according to the manufacturer’s instructions. The SOD, CAT, and GSH-Px activities were determined using a microplate reader (Multiskan Spectrum, Thermo) at 450 nm, 405 nm, and 412 nm, respectively.

### Assessment of mitochondrial lipid peroxidation

The extraction and concentration determination of mitochondrial protein were performed according to the above description. Lipid peroxidation was assessed by MDA concentration using an MDA assay kit (A003-1, Nanjing Jiancheng Bioengineering Institute, China). The MDA level was determined by the thiobarbituric acid method and was measured at 535 nm using a microplate reader (Multiskan Spectrum, Thermo).

### TMT-based quantitative proteomic analysis

The spermatozoa samples under liquid storage at 0 h (group A), 48 h (group B), and 96 h (group C) were used for quantitative proteomic analysis using three biological replicates. Protein was extracted from the stored spermatozoa in lysis buffer (8 M urea, 2 mM EDTA, and 1% Protease Inhibitor Cocktail) using a high-intensity ultrasonic processor (Scientz Biotechnology, Ningbo, China), and the protein concentration was determined using the BCA kit according to the manufacturer’s instructions. The samples were then subjected to trypsin digestion, TMT labeling, HPLC fractionation, and LC-MS/MS based on the previously reported methods [66]. The peptide sample was labeled using the 10-plex TMT kit (Thermo Fisher Scientific, Rockford, IL) according to the manufacturer’s protocol. The labeled peptides were then fractionated using high-pH reverse-phase HPLC (Agilent 300 Extend C18 column; 5 μm particles, 4.6 mm ID, and 250 mm length). The graded sample was separated using the Easy-nLC 1000 UPLC system (Thermo Scienctific) and then was subjected to the NSI source followed by MS/MS in Q Exactive^TM^ Plus (Thermo Scienctific) coupled online to the UPLC. The raw MS/MS data were processed using the Maxquant search engine (v.1.5.2.8). Tandem mass spectra were searched against the *Capra hircus* protein database in NCBI (42,687 sequences). The proteomic data have been deposited in ProteomeXchange via the PRIDE database with the accession number PXD014609.

### Bioinformatics analysis

R package was used for the analyses of PCA and PCC to detect the variations between different replicated protein samples. The protein false discovery rate (FDR) was adjusted to < 1%, and the minimum score for peptides was set at > 40. Only protein with a fold change > 1.5 (or < 0.67) and a *p*-value < 0.05 was considered to be DEPs between two sample groups. Protein functional annotation and classification were conducted according to GO and KEGG databases. Enrichment analysis of DEPs was further using a corrected *p*-value < 0.05 to determine a significant difference. GO annotation and classification were performed according to three major categories: biological process, cellular component, and molecular function. KEGG pathway annotation was performed using KEGG online service tools. A heat map of protein expression was generated by using MultiExperiment Viewer (MeV) software based on the values of fold change. Online STRING (version 10.5) software was employed for protein-protein interactions with a confidence score > 0.4.

### Statistical analysis

The data are presented as the means ± standard error of mean (SEM). Statistical analysis was conducted with SPSS 16.0 software (SPSS, Inc., Chicago, IL, USA), and significance was assessed by one-way ANOVA. Statistical significance was defined as *P* < 0.05.
